# Cardioprotective Roles of β-Hydroxybutyrate Against Doxorubicin Induced Cardiotoxicity

**DOI:** 10.3389/fphar.2020.603596

**Published:** 2021-04-15

**Authors:** Yihai Liu, Xuan Wei, Mingyue Wu, Jiamin Xu, Biao Xu, Lina Kang

**Affiliations:** Department of Cardiology, Nanjing Drum Tower Hospital as Affiliated Drum Tower Hospital, Nanjing, China

**Keywords:** β-hydroxybutyrate, doxorubicin, cardiotoxicity, heart failure, metobolites

## Abstract

**Background:** β-Hydroxybutyrate (BHB) is produced by fatty acid oxidation in the liver under the fasting state and confirmed to play a cardioprotective role in ischemia and hypertensive settings. Doxorubicin (DOX) is an effective chemotherapeutic drug, but limited by serious irreversible cardiotoxicity. However, whether BHB can protect from DOX-induced cardiotoxicity remains unknown.

**Methods and Results:** C57BL/6 mice were intraperitoneally injected with DOX to induce cardiac toxicity and intragastrically administered into BHB for treatment. They were randomly divided into three groups, namely a sham group (Sham), a doxorubicin group (DOX), and a doxorubicin+β-Hydroxybutyrate group (DOX + BHB). Echocardiography and pathological staining were performed to evaluate cardiac function and fibrosis. H9c2 cardiomyocyte was treated with DOX or BHB for *in vitro* experiments. Cell apoptosis and ROS were determined by flow cytometry. BHB significantly restored DOX-induced cardiac function decline and partially prevented cardiac reverse remodeling, characterized by increased cell size and decreased fibrosis. *In vitro*, BHB treatment decreased cellular injury and apoptosis. Also, BHB alleviated oxidative stress level and increased mitochondrial membrane potential.

**Conclusion:** Our results suggested that BHB could protected from DOX-induced cardiotoxicity by inhibiting cell apoptosis and oxidative stress and maintaining mitochondrial membrane integrity.

## Introduction

Doxorubicin (DOX) is widely recognized as antineoplastic for the treatment of several malignant tumors including solid tumors, leukemia and lymphoma (Minotti et al., 2004). However, its clinical use is limited by severe cardiotoxic side effects, including arrhythmia, dilated cardiomyopathy and subsequent heart failure (Octavia et al., 2012). DOX-induced cardiac injury had poor prognosis and high mortality (Wouters et al., 2005). Researchers over the past two decades had demonstrated that ROS production (Liu et al., 2020), cell apoptosis (Yu et al., 2020) and autophagy (Sun et al., 2014; Ma et al., 2017), DNA damage (Mizuta et al., 2020), mitophagy (Liang et al., 2020; Xu et al., 2020) and various signaling pathways (Chen et al., 2020) were the potential mechanisms, providing further insight into treatment strategies to prevent the DOX-induced cardiotoxic effects. Meanwhile, it was no effective to utilize various kind of antioxidant agents to suppress oxidative stress in the clinical trials. To date, no treatment is unanimously recommended for attenuating DOX-induced cardiotoxicity, indicating the need for further investigations (Varricchi et al., 2018).

β*-*Hydroxybutyrate (BHB), derived from increased free fatty acid β*-*oxidation*,* is small lipid-derived molecules (Veech et al., 2001). In addition, it is known to play an immunomodulatory role via ligating membrane receptor Hcar2 (Youm et al., 2015), inhibiting inflammasome activation and histone deacetylase expression (Chang et al., 2014). BHB had been reported to protect against tissue injury via decreasing the production of reactive oxygen species and attenuating mitochondrial injury. Considering that oxidative stress and mitochondrial injury largely contributed to DOX-induced cardiac injury, we speculated BHB could modulate DOX related cardiotoxicity.

To date, some studies have verified the beneficial hemodynamic effects of BHB in heart failure with reduced ejection fraction and other age-associated disease (Han et al., 2020). However, it’s unknown whether BHB can be envisioned as a promising novel metabolite to treat DOX-induced cardiotoxicity. In our study, we evaluated the cardioprotective effect of BHB on DOX-induced cardiotoxicity *in vitro* and *in vivo*.

## Methods

### Animals

After 1 week of adaptation to the housing environment, adult male C57/BL6 mice (6–8 weeks old, Institute of Model Animals of Nanjing University) were randomly divided into three groups: Sham group, DOX group, and DOX + BHB group. The doxorubicin (Sigma, United States) was injected intraperitoneally into mouse at a dose of 5 mg/kg/day for 5 days to induce cardiac injury. In DOX + BHB group, -Hydroxybutyrate (Sigma, United States) was injected into mice at a dose of 10 mmol/kg/day via 5 daily intraperitoneal injections. The Sham group was injected intraperitoneally with the same volume of saline as control. Each group has six mice. All procedures have been approved by Institutional Animal *Care* and Use Committee (IACUC) of Nanjing Drum Tower Hospital.

### Echocardiography

At the fifth day, all animals underwent echocardiography (Visual Sonics; Japan) to evaluate the cardiac function. In briefly, mice were anesthetized by inhalation of 1.5% isoflurane, and shaved. The parameters, including left ventricular internal diameter end systole (LVIDs) and left ventricular internal diameter end diastole (LVIDd), were measured and averaged from three consecutive cardiac cycles. Ejection fraction (EF) and fractional shortening (FS) were calculated accordingly.

### Cell Culture and Treatment

The H9c2 cell line was purchased from American Type Culture Collection (ATCC) and cultured in Dulbecco’s modified Eagle’s medium with 10% fetal bovine serum and 1% streptomycin and penicillin (Gibco; United States). The culture conditions contained 95% air and 5% CO_2_ at 37°C. DOX group was incubated with Dox (1 μM) for 24 h while DOX + BHB group was also treated with extra BHB (10 mM) for 24 h.

### Lactate Dehydrogenase Assay

After cultured for 24 h, 100 μl of cell supernatants of each group were collected. Lactate dehydrogenase (LDH) release level was assessed by using a commercial kit (Keygen; China) following the manufacturer’s instructions. The experiments were performed in triplicates.

### TUNEL Assay

Terminal deoxynucleotidyl transferase dUTP nick end labeling (TUNEL) staining was used to evaluate cardiac apoptosis according to the manufacturer’s instructions (Servicebio; China). Apoptosis was indicated as the ratio of TUNEL-positive nuclei in three images per heart.

### Morphological Analysis

Mice were sacrificed by cervical dislocation after anesthesia with tilotamine (0.09 mg/g), zolazepam (0.09 mg/g), and 0.01% xylazine (0.04 mL/g); and then their heart received a KCL injection in order to stop the heart in diastole. Heart from each group was excised and washed in cold PBS, fixed overnight in 10% formalin, and finally embedded in paraffin blocks. The slices were stained with hematoxylin-eosin and Sirius red (Servicebio; China) to evaluate cardiac remodeling. Cell area and interstitial fibrosis were quantified by Image J (NIH; United States).

### Quantitative Real-Time Polymerase Chain Reaction

Total RNA was extracted from cardiac tissues using RNAiso plus (TaKaRa; Japan) according to the manufacturer’s instructions. 1 μg of RNA was reverse transcribed into cDNA with HiScriptII Q RT SuperMix (Vazyme; China) and quantitative RT-PCR was performed with ChamQ SYBR qPCR Master Mix (Vazyme; China) according to the protocol. All the results were normalized against glyceraldehyde-3-phosphate dehydrogenase (GAPDH) expression.

### Measurement of Mitochondrial Membrane Potential

Mitochondrial membrane potential was stained with 5,5′,6,6′-tetrachloro-1,1′,3,3′-tetraethylbenzimidazol -carbocyanine iodide kit (JC-1) (Keygene; China). The primary cardiomyocytes were seed in a 96-well pates and subjected to treatment. After washed by PBS, they were stained with JC-1 solution and visualized using a fluorescence microscopy (Leica; Germany). All procedures the the manufacturer’s instructions and protocol.

### Malondlaldehyde Measurement

The cardiac tissue was thawed at room temperature and washed with precooled normal saline (NS). Homogenates were centrifuged (10 min at 1500 g, 4°C) to eliminate debris, and the resulting supernatant was stored at liquid nitrogen (−80 C) until analysis. MDA was estimated using the detection kit (Jiancheng Bio-Technology; China) according to the manufacturer’s instructions.

### Western Blotting

Total protein was extracted from heart tissues using a RIPA buffer containing proteinase and phosphatase inhibitor (Roche; Germany). After determining the protein concentrations using a BCA kit (Thermo; United States), protein lysates were separated by SDS-PAGE and transferred to PVDF membranes (Millipore; United States). The membranes were incubated with the corresponding primary antibodies, such as ERK1/2, phosphor-ERK1/2, MMP9, Nrf2 and GAPDH (Abcam; United States) overnight at 4°C before being incubated with goat anti-rabbit lgG (Abcam; United States). The western blot bands were detected using ECL kit (Keygene; China). For quantification by imageJ (NIH; United States), the specific protein expression levels were normalized to GAPDH levels.

### Statistical Analysis

GraphPad Prism 8.0 (GraphPad; United States) was used to plot the figures based on the data presented as mean ± SD. Statistical analysis was done using one-way ANOVA, followed by Tukey’s pos hoc test using IBM SPSS 25.0. *p* value < 0.05 was considered as statistically significant.

## Results

### BHB Prevented Doxorubicin-Induced Left Ventricular Dysfunction

**FIGURE 1:**
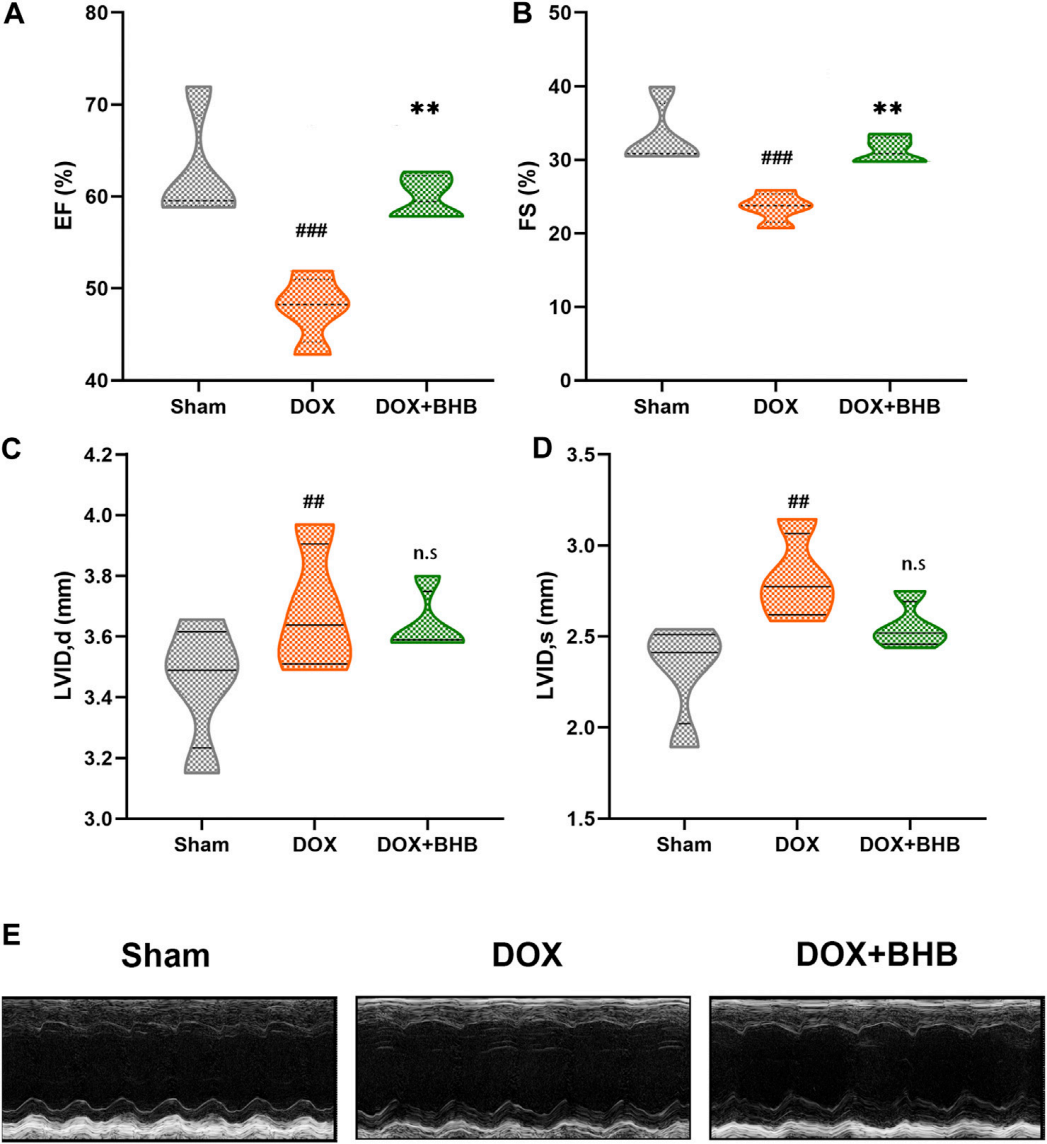
BHB prevents doxorubicin-induced left ventricular dysfunction. **(A)** Ejection fraction (EF); **(B)** fractional shortening (FS); **(C)** left ventricular diastolic internal dimension (LVIDd); **(D)** and left ventricular systolic internal dimension (LVIDs) among sham, DOX and DOX + BHB groups; **(F)** the presentative echocardiography images among groups. Each group had 5 mice. ##*p* < 0.01, ###*p* < 0.001 vs. Sham. **p < 0.01 vs. DOX group. n. s, not significant.

At the terminal of the treatment, echocardiography was performed to measure cardiac function. As expected, DOX-treatment led to a significant decrease in ejection fraction (EF). Compared with the DOX-group, EF was higher in DOX + BHB group (Figure 1A). Similarly, fractional shortening (FS) was decreased in DOX-group but was restored by BHB intervention (Figure 1B). Besides, BHB attenuated the adverse cardiac remodeling as represented by reduced left ventricular internal diameter end diastole (LVIDd) and left ventricular internal diameter end systole (LVIDs) (Figures 1C,D). However, the difference was not statistically significant, which could be attributed to a short course. The representative echocardiographic images were shown in Figure 1E.

### BHB Protected from DOX-Induced Cardiac Remodeling

**FIGURE 2:**
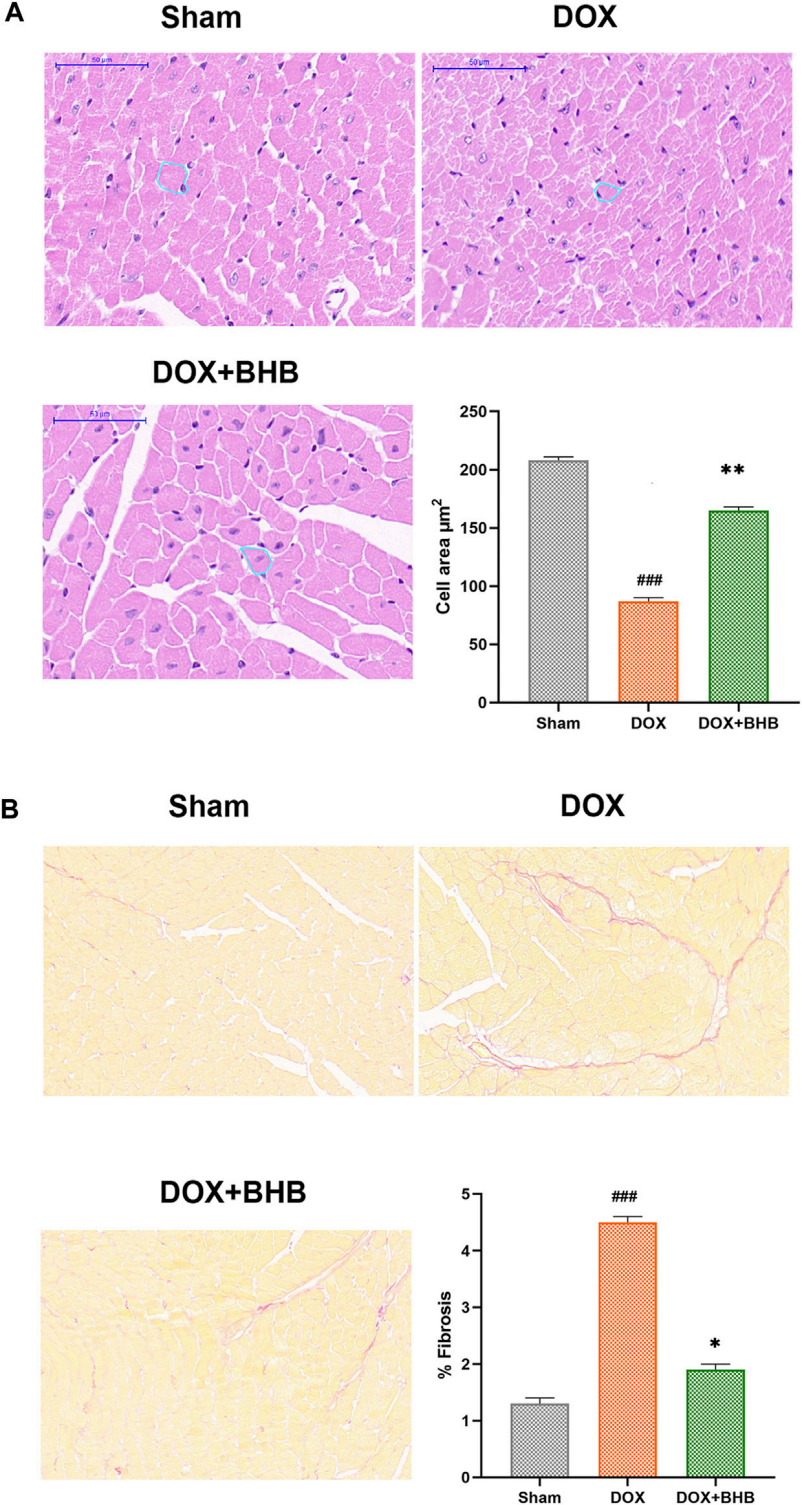
BHB protects from DOX-induced cardiac remodeling. **(A)** HE staining; **(B)** Sirius red of tissues from sham, DOX and DOX + BHB group. Each group had 5 mice. ###*p* < 0.001 vs. Sham; *p < 0.05, **p < 0.01 vs. DOX.

DOX-induced cardiotoxicity was characterized by cardiac fibrosis and reduced cardiomyocyte size. Our study results revealed that BHB restored the reduction in cardiomyocyte size induced by DOX (Figure 2A). What’s more, BHB mitigated the percentage of interstitial fibrosis (Figure 2B).

### BHB Inhibited Cell Apoptosis Induced by DOX

**FIGURE 3:**
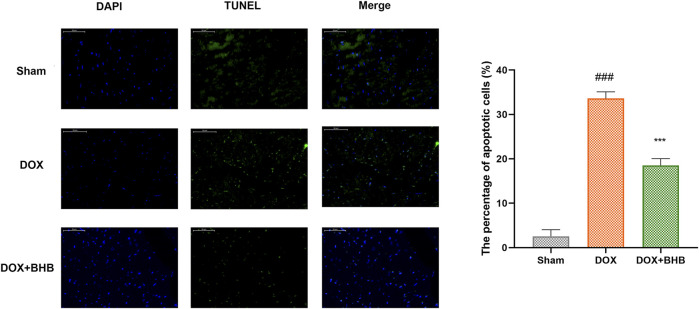
BHB prevents DOX-induced cardiac cell apoptosis. Blue indicates nucleus, green indicates TUNEL positive cells, and Merge for apoptotic cells. Each group had 3 samples. ###*p* < 0.001 vs. Sham; ***p < 0.001 vs. DOX.

TUNEL staining was performed to determine whether BHB played an antiapoptotic role in DOX induced cardiac injury. As shown in Figure 3, BHB significantly decreased the percentage of TUNEL positive cells compared with DOX group.

### BHB Alleviated Oxidative Stress Induced by Doxorubicin

**FIGURE 4:**
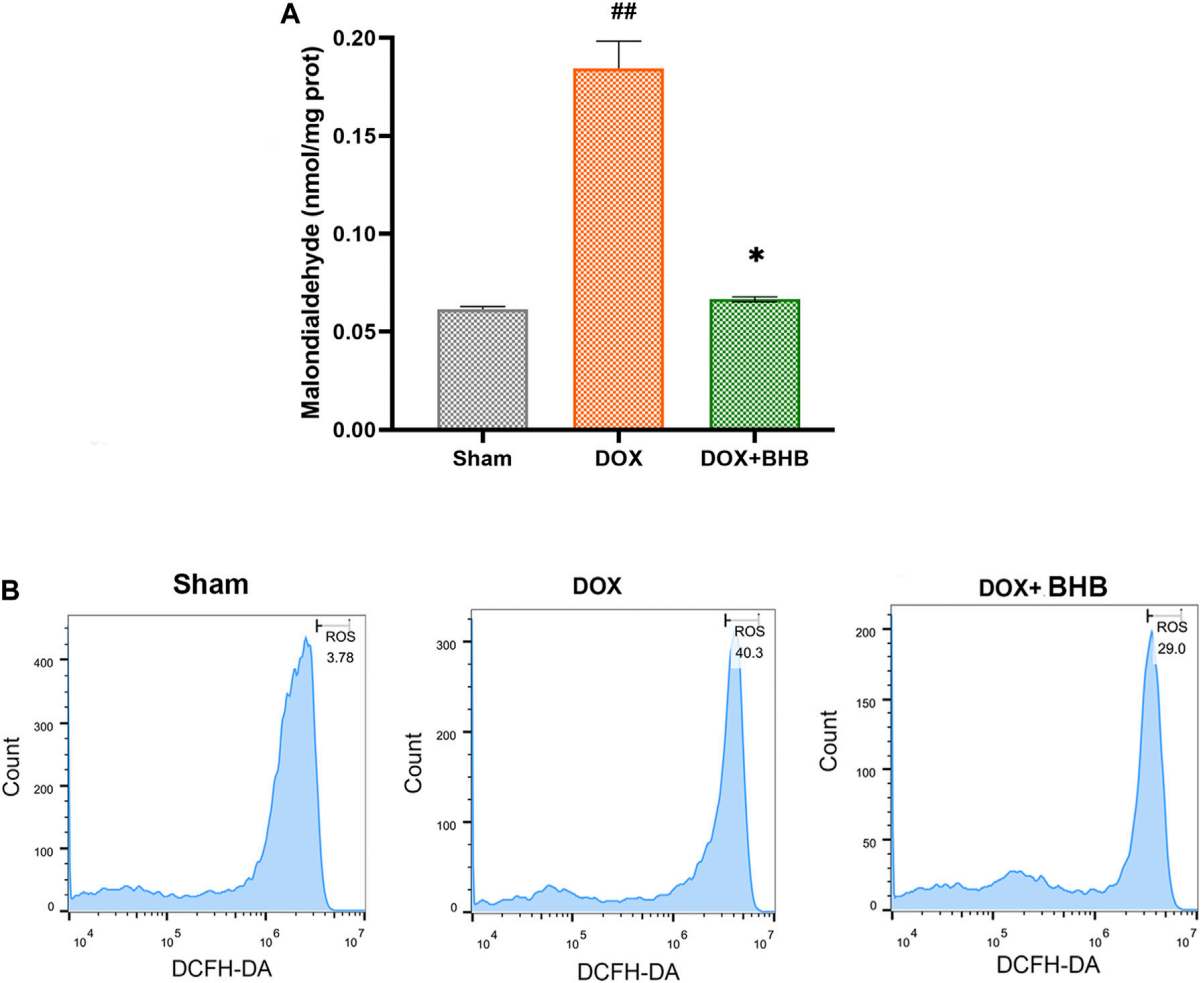
BHB alleviates oxidative stress induced by doxorubicin. **(A)** The MDA level among groups. **(B)** The ROS detection of cardiomyocytes among groups. Each group had 3 samples. ##*p* < 0.01 vs. Sham; *p < 0.05 vs. DOX.

Oxidative stress is an important aspect of Dox-induced cardiac injury, so we speculated that BHB could alleviate Dox-induced oxidative stress. Comparing with DOX group, DOX + BHB group had a lower level of MDA, suggesting an anti-oxidative stress effect of BHB (Figure 4A). Consistent with the above results, flow cytometry showed that BHB significantly inhibited ROS level induced by doxorubicin (Figure 4B).

### BHB Counteracted Mitochondrial Membrane Potential Decrease Induced by Doxorubicin

**FIGURE 5:**
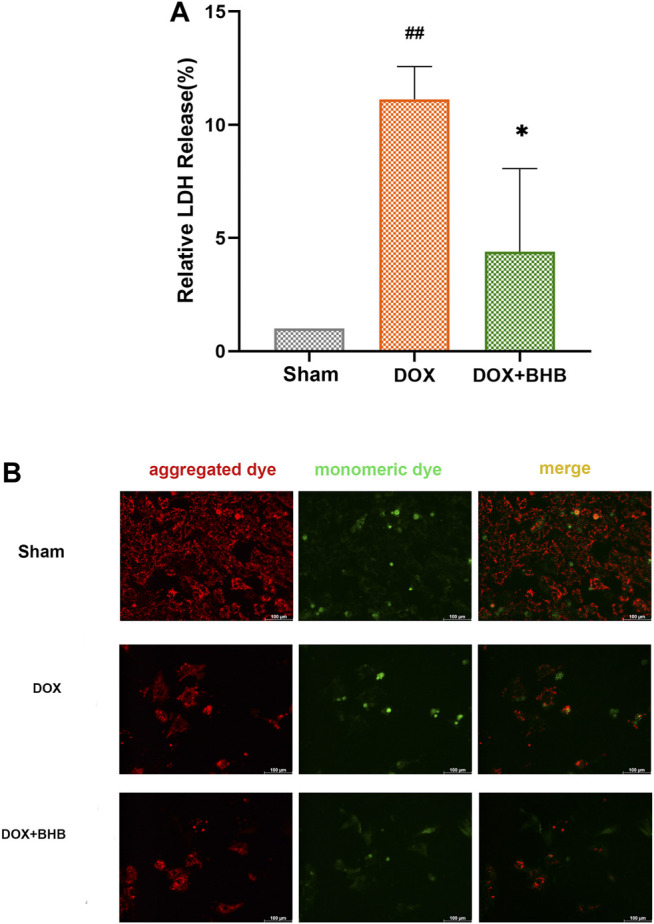
BHB counteracts mitochondrial membrane potential decrease induced by doxorubicin **(A)** The LDH release level among groups. **(B)** The JC-1 staining of different groups. Red fluorescent signal (aggregated dye) indicated a normal mitochondrial membrane potential while green fluorescent signal (monomeric dye) suggested a decreased mitochondrial membrane potential. Each group had 3 samples. ##*p* < 0.01 vs. Sham; *p < 0.05 vs. DOX.

H9c2 cardiomyocytes were treated with BHB to investigate its cardioprotective role. DOX could cause H9c2 cardiomyocyte injury, indicated by increased LDH release (Figure 5A), while BHB decreased LDH release. DOX treatment decreased the ratio of red (aggregated dye) to green fluorescent signal (monomeric dye), indicating a downregulation of mitochondrial membrane potential. However, BHB prevented DOX-induced decrease in the JC-1 aggregate/monomer ratio, implying that BHB may contribute to maintaining mitochondrial integrity (Figure 5B).

### BHB Downregulated MMP9 Expression

**FIGURE 6:**
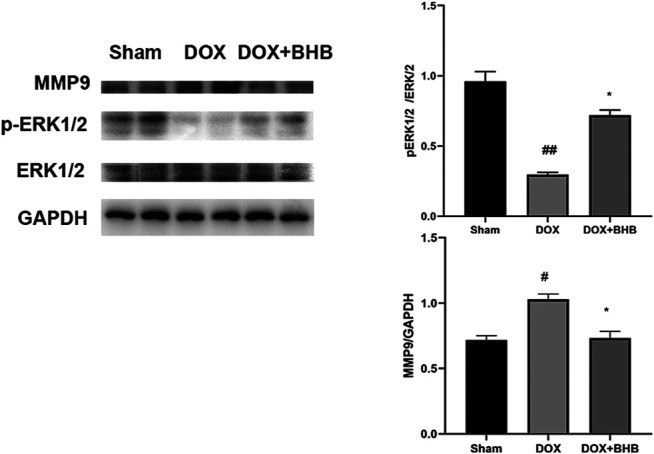
BHB alleviated cardiac injury via modulating ERK1/2 phosphorylation and MMP9 expression. The up-left is immunoblotting bands while two else are quantitative results. Each group had 2 samples. #*p* < 0.05 vs. Sham; *p < 0.05 vs. DOX.

To investigate the downstream pathway, we performed immunoblotting analysis of BHB-treated hearts. The ERK1/2 phosphorylation was decreased and MMP9 expression were increased in DOX group while BHB restored the expression level of ERK1/2 phosphorylation and MMP9 (Figure 6), suggesting that activating ERK1/2 pathway could mediate the cardioprotective role of BHB.

## Discussion

In this study, we showed for the first time that BHB could prevent DOX-induced cardiotoxicity by inhibiting oxidative stress, cell apoptosis and maintaining mitochondrial integrity *in vitro* and *in vivo*, partially restoring cardiac function.

β*-*Hydroxybutyrate (BHB), one of ketone body, has been traditionally regarded as an alternative carrier of energy (Zhang et al., 2013). Besides, BHB has been proven beneficial in cardiovascular diseases (Han et al., 2020). Previous studies had proposed some strategies for DOX-induced cardiac injury. Li et al. demonstrated that Sestrin1/2 were important cardioprotective proteins to minimize cardiac damage in response to doxorubicin insult (Li et al., 2019). Ivabradine (El-Naggar et al., 2018) and some traditional Chinese medicine (Xu et al., 2018) also played a cardioprotective role in DOX induced cardiotoxicity. We confirmed that BHB could be a cardioprotective metabolite in the future.

Among the widely studied mechanisms underlying DOX related cardiotoxicity, one of the most accepted is oxidative stress induced by free radicals (Hadi et al., 2012). Free radicals can cause membrane and macromolecules damage and lead to myocardial damage directly (Kim et al., 2006). DOX can destabilize the delicate balance between the generation of free radicals and the antioxidant defense system (Hadi et al., 2012). In addition to its metabolic effect, BHB also played an immunomodulatory role via regulating inflammation and oxidative stress (Nagao et al., 2016; Kong et al., 2017). Some reports also found that BHB could provide mitochondria protection (Zhang et al., 2013; Kim et al., 2015). In according to previous publications, we also confirmed that BHB could prevent from DOX-induced oxidative stress and cardiomyocyte apoptosis.

In line with our results, BHB have also been shown to protect against ischemic tissue injury when present at low concentrations, including cerebrum (Bazzigaluppi et al., 2018), heart (Suzuki et al., 2001), liver (Chen et al., 2018), and kidney (Tajima et al., 2019), mainly through decreasing the production of reactive oxygen species and attenuating mitochondrial injury (Yu et al., 2018). It has been reported that increased oxidative stress promoted matrix metalloproteinase-9 (MMP-9) activities and may lead to cardiomyocyte injury (Sousa-Santos et al., 2012). Oxidative stress-induced damage can be attenuated potentially through the ERK1/2 pathway (Zhu et al., 2019). Activation of ERK1/2 pathway could protect cells from oxidative injury via activating and phosphorylating several downstream transcription factors such as AP-1, c-Jun and c-Myc for cell survival (Plotnikov et al., 2011). Specially, ERK1/2 activated Elk1 and increased the transcription of superoxide dismutase (Wang et al., 2011). ERK1/2 also induced nucleus translocation of Nrf2 (Li et al., 2018). Overall, our findings revealed that ERK1/2 signaling modulation may mediate the cardioprotective role of BHB.

Our study had proven that BHB could prevent DOX-induced adverse cardiac remodeling. Specially, BHB can inhibit the oxidative stress and cardiomyocyte apoptosis by sustaining mitochondrial membrane potential integrity. The potential clinical application of BHB in DOX-treatment cancer patients deserved to be further explored.

## Data Availability

The raw data supporting the conclusions of this article will be made available by the authors, without undue reservation.
